# Asthma in the Elderly: Can We Distinguish It from COPD?

**DOI:** 10.1155/2011/843543

**Published:** 2011-06-30

**Authors:** Eleni G. Tzortzaki, Athanasia Proklou, Nikolaos M. Siafakas

**Affiliations:** Department of Thoracic Medicine, University Hospital of Heraklion and Medical School, University of Crete, Heraklion 71110, Crete, Greece

## Abstract

Asthma in older adults affects quality of life and results in a higher hospitalization rate and mortality. In common clinical practice, asthma in the elderly is underdiagnosed and undertreated or overdiagnosed and mistreated. The age-related reduction in perception of shortness of breath and the high incidence of comorbidities make the diagnosis and management more difficult and challenging for the physicians. Chronic obstructive pulmonary disease (COPD) is usually easy to distinguish from asthma, but sometimes the distinction from late-onset asthma in older patients, particularly in cigarette smokers, is difficult and may be impossible. Both diseases are characterized by the presence of airflow obstruction but have distinct pathogenesis, inflammatory pattern, and prognosis. The distinction between Asthma and COPD based simply on spirometric parameters is difficult especially in the elderly asthmatics. The combination of lung function testing, bronchial hyperresponsiveness (BHR) and atopy status, HRCT scans, and the newly developed biological techniques, allowing the assessment of biomarker profiles, could facilitate the distinction between these diseases.

## 1. Introduction

The increasing burden of obstructive lung diseases, such as Asthma and Chronic Obstructive Pulmonary Disease (COPD), appears to be caused, at least in part, by the ageing of the world's population [1]. The World Health Organization estimates that between the years 2000 and 2050, the proportion of persons over 65 years of age is expected to represent up to 17% of the total world population [1]. In the case of asthma, the prevalence in the elderly is also high, affecting >10% of patients >60 years of age, while the estimated prevalence for COPD represents a 20% to 30% in patients >70 years of age [2, 3].

Although it was believed that asthma is predominantly a childhood disease [4, 5], asthma in the elderly is not a rare disorder [6]. Moreover, it is frequently underdiagnosed or misdiagnosed [4, 5] due to atypical presentation, age-related reduction of dyspnea perception, and associated comorbidities [6, 7]. 

In particular, Asthma and COPD may overlap and converge in older people [6, 8]. Ageing is associated with unique issues that modify expression, recognition, and treatment of the disease [6, 7]. Many older adults with asthma have a persistent airway obstruction on pulmonary function testing which makes it difficult to differentiate asthma from COPD [8–11]. Both diseases are associated with similar symptoms such as shortness of breath, wheezing, and coughing with or without sputum production [6, 8]. Furthermore, to distinguish COPD from late-onset asthma, particularly in elderly smokers, often remains a challenge for the clinician [6, 8]. For example, a longitudinal study found that 16% of patients with asthma developed features consistent with irreversible airflow obstruction after 20 to 30 years of followup [9].

Nevertheless, Asthma and COPD are two quite different and independent diseases with different pathogenesis, different pathology, different type of inflammation, and different prognosis [8] (Table 1). Asthma traditionally characterized by an eosinophilic inflammation affects all the airways but not lung parenchyma, and it is linked to airway hyperresponsiveness [4, 5]. The airflow obstruction in asthma is often reversible spontaneously or with treatment [4, 5]. On the other hand, COPD refers to a disorder in which airflow limitation is not fully reversible [12]. The airflow obstruction is caused by damage to the small airways as a result of airway inflammation (predominantly neutrophilic), structural changes in small and large airways (remodelling), and parenchymal destruction as a result of loss of alveolar attachments and decrease in elastic recoil (emphysema) [8, 12]. Although the clinical features of these diseases help us to differentiate between them in most patients, the spectrum of obstructive lung diseases seen in clinical practice is more complex, particularly, among the elderly, who may have components of both diseases [8].

The aim of this paper is to highlight the main and more useful “tools” for Asthma and COPD differentiation in the elderly.

## 2. Approaching Elderly Asthmatics: Recognition of Symptoms

Elderly patients with suspected asthma require a systematic approach to their diagnosis [13, 14]. Older patients with asthma underestimate the severity or significance of their symptoms, attribute symptoms to other causes such as ageing, COPD, or cardiac failure, or ignore symptoms entirely. For example, breathlessness, a common symptom affecting up to a third of older people, often is perceived to be part of the normal process of ageing [13–15]. Self-limitation of activities, social isolation, and depression further contribute to underdiagnosis of asthma in older adults [15]. The condition that presents the greatest diagnostic confusion for asthma among older persons is COPD [13, 14]. 

Although asthma and COPD share symptoms, their presentation is typically different [4, 5, 12]. Symptoms such as wheezing, breathlessness, chest tightness, and coughing should be carefully evaluated. Older persons with COPD often experience breathlessness when they exert moderate or even minimal physical efforts. On the contrary, breathlessness in asthma usually occurs in exacerbations [11, 13]. Coughing, which can be the only presenting symptom among older persons with asthma, is also associated with an exacerbation, while it occurs regularly with COPD [11, 13]. 

Conversely, the differentiation of symptoms is not always as straightforward as the above examples. Wheezing, the most common symptom of asthma in the elderly [13, 14], can be also attributed to other pathological conditions such as COPD, cardiac failure, acute bronchitis, bronchiectasis, gastroesophageal reflux, aspiration or inhalation of foreign body, and tracheobronchial tumors [10, 11, 16]. Breathlessness is usually perceived as part of the normal process of ageing. Clinicians have to rule out other diseases such as COPD, congestive heart failure, pulmonary embolism, hyperventilation/panic disorder, Churg-Strauss syndrome, and other vasculitis [10, 11]. 

## 3. Spirometry, Pulmonary Function Tests and Bronchial Hyperresponsiveness (BHR)

Pulmonary testing with bronchodilators has been used to differentiate asthma from COPD, with COPD obtaining a lesser response [4, 5, 12]. A final diagnosis of asthma is determined when either clinic spirometry or pulmonary function tests demonstrate airflow obstruction that improves significantly, defined as both a 12% and 200 mL improvement in forced expiratory volume in one second (FEV1) in response to inhaled bronchodilator [4, 5]. However, previous studies demonstrated that an increase ≥10% of the predicted FEV1 after inhalation of a short-acting bronchodilator may have a higher likelihood of distinguishing asthmatics from COPD patients [17]. To further confuse the distinction between asthma and COPD, a recent study with over 5000 COPD patients found that more than two-thirds showed meaningful increases in lung function following administration of bronchodilators [18].

Nonetheless spirometry, before and after bronchodilation, should be performed although it may have some additional difficulties in the elderly. Several studies reported that spirometry can be adequately done in the majority of older patients when the staff is properly trained and with the appropriate application of reference values in this age group [16, 17]. The American Thoracic Society/European Respiratory Society pulmonary function test interpretation guidelines recommend that the lower limit of the normal range for FEV1/FVC ratio, based on the fifth percentile corrected for age, sex, height, and race, be used to detect airflow obstruction [17].

The fixed airflow limitation of most patients can be defined as a postbronchodilator FEV1 of <80% pred (in the presence of a reduced FEV1/FVC) after a 7-to-14 day course of oral corticosteroids (40 mg per day for adults) [19]. A negative response may indicate COPD or rarely refractory asthma (corticosteroid resistant) [20, 21]. Population studies have shown that as many as 30% of patients with fixed airflow obstruction have a past history of asthma [22]. 

Often times there is a need for more tests such as lung volumes (total lung capacity (TLC), functional residual capacity (FRC), residual volume (RV), RV/TLC%), or diffusing capacity (DLCO) of the lung. Lung volumes are elevated in COPD [12]. The presence of a normal diffusing capacity for carbon monoxide (DLCO) can be useful to differentiate patients with asthma from patients with COPD; nevertheless, patients with asthma and a history of smoking may also present a reduced DLCO [16, 21]. Generally, the decreased DLCO may be directly related to the loss of alveolar-capillary surface area that is associated with emphysema [12, 17, 21].

Bronchial hyperresponsiveness (BHR) has long been considered a differentiating feature of asthma [4, 5]. The role of BHR in the elderly is a matter of debate. Scichilone et al. in a review of eighteen studies showed a positive association between age and airway hyperresponsiveness, the prevalence of which appears to increase in the elderly [23]. The most important determinants were reduced lung function, probably due to geometric factors, and a history of smoking. Atopy, female sex, inflammatory, and neuronal mechanisms should also be considered as determinants of airway hyperresponsiveness in the elderly [23]. 

Likewise, early detection of airway hyperresponsiveness in these individuals could permit the identification of elderly asthmatics at an earlier stage so as to optimize the treatment of this disease [23, 24]. However, the physician needs to be alert during BHR tests since the airways of elderly patients with asthma are more reactive than those of young asthmatics when airway responsiveness is expressed in terms of methacholine-induced changes in FVC [25]. This implies that elderly asthmatic patients are at higher risk of airway closure. Furthermore, elderly individuals experience less awareness of bronchoconstriction during methacholine bronchoprovocation, despite similar degrees of bronchoconstriction. The presence of one or more co-morbid conditions, which become common in the elderly, significantly impair perception of bronchoconstriction, and the condition of unrecognised airway hyperresponsiveness could lead to the occurrence of severe airway narrowing [14–16].

The role of BHR as a risk factor for obstructive lung diseases in the elderly has not been well studied, although reports have shown that airway hyperresponsiveness may be more common than previously thought among older adults [26, 27]. In COPD patients, the Lung Health Study found BHR to methacholine in 63% of men and 87% of women [26]. The authors stated the hypothesis that an important determinant of BHR is gender differences in airway caliber and smoking. In conclusion, measuring BHR might be useful also in patients with COPD. However, to establish these methods in clinical routine in COPD patients, more data are clearly needed [27].

## 4. Structural Changes

Asthma in an older adult is the result either of childhood asthma that is long-lasting, or late-onset disease [28]. Age-related changes lead to increased air trapping and a reduction in chest wall compliance. Degenerative changes in muscles cause reduced force generation capacity of the respiratory muscles and an increased work of breathing [6, 10, 11, 29]. 

In general, asthma in older adults is characterized by less IgE-mediated processes and more frequently by irreversible airway obstruction. An accelerated decline in lung function occurs in elderly asthmatics, mainly due to airway remodelling, especially in the small airways from inflammation that characterizes asthma [28, 29]. Compared with the general population, elderly asthmatics are more likely to die of lung diseases, presumably due to respiratory tract infections, especially those with poor socio-economic status [29, 30].

In detail, airflow obstruction in asthma is the results of a serial of structural changes of the airway lumen rather than changes in lung parenchyma [31–33]. The CD4^+^ Th2 lymphocytes may have important role in maintaining this specific asthmatic airway inflammatory cascade [31–33]. Chronic inflammation in asthma results in bronchial remodelling characterized by basement membrane thickening, mucosal blood vessels proliferation, extracellular matrix proteins deposition, mucus gland stimulation, smooth muscle cell and myofibroblast proliferation, and finally defective epithelium regeneration and atrophy [31–33]. While some of these changes may be reversible with treatment, at some point, they may become permanent causing fixed airway obstruction. In elderly asthmatics the presence of emphysema is minimal, and airway remodeling is thought to be the main cause of fixed airflow obstruction [31–33]. 

On the contrary, in COPD it seems that fixed airway obstruction is the final result of structural changes of peripheral airways and the lung parenchyma [31–33]. Infiltration of the airways with neutrophils and CD8(^+^) lymphocytes in COPD is associated with airway remodeling, mucous plugging, smooth muscle hypertrophy, and airway wall fibrosis [31–33], (Table 1). In COPD, the remodeling of the small airways and lung parenchyma are the main correlates to the limitation of expiratory airflow [32–35]. Thickening of the airway wall, goblet cell hyperplasia, mucous gland hypertrophy, and the luminal obstruction caused by inflammatory exudates and mucous are features of COPD. Squamous epithelial metaplasia and airway wall fibrosis are commonly observed characteristics of COPD [32–35]. Destruction and fibrosis of the alveolar wall occur in COPD but not in asthma [32–35]. 

Recently, *High-resolution CT scan (HRCT)* has been proposed as an additional tool to assess pulmonary structural changes in long-standing diseases, such as asthma and COPD. Moreover, by differentiating airway remodelling from parenchymal alterations, HRCT imaging could contribute towards distinguishing between asthma and COPD [36, 37]. Anatomical pulmonary changes, including bronchial wall thickening, emphysema, and bronchiectasis, have been demonstrated by HRCT scan in some studies [36–38]. HRCT scan has been previously used to quantify abnormalities of the airways due to airway remodeling, and it has been found that the HRCT scan score correlated with the severity of asthma and airflow obstruction [36–38]. However, there are no studies so far evaluating specifically abnormalities in lung CT of elderly asthmatics. In a recent study [39], it was assessed whether airway remodelling is restricted to specific subphenotypes of severe asthma. Patients included were between 43–54 years old, and the results showed that the degree of airway wall remodelling assessed by CT scanning was similar in all severe asthma subphenotypes and was associated with airflow limitation and neutrophilic inflammation [39]. To summarize, the above studies demonstrated a close relationship between HRCT scan alterations and the extent of in vivo remodelling occurring in the airways of patients with obstructive airway diseases. The use of high-resolution computed tomography imaging could contribute to differential diagnosis between asthma and COPD.

Lately, *high-dimensional biological techniques* (e.g., genomics, metabolomics) allow assessment of disease biomarker profiles [40]. This provides opportunities to discriminate disease entities based on composite molecular signatures. This may not only be applicable to serum or bronchoalveolar lavage fluid but also to noninvasive alternatives such as exhaled air and sputum [40, 41]. Exhaled air is known to contain thousands of volatile organic compounds (VOCs) that are derived from various metabolic and inflammatory pathways in the lung [41]. Fens et al. [41] showed that “breathprints” from patients with asthma were different from patients with COPD (accuracy 96%), from nonsmoking control subjects, and from smoking control subjects [41]. 

Furthermore, *induced sputum* is an extensively used noninvasive method that can serve as a window to assess the microenvironment of the lungs. The first studies that revealed somatic mutations in the form of Microsatellite DNA Instability (MI), in sputum cells, were in COPD [42, 43] and later on in Asthma [44]. However, in a comparative study between COPD and Asthma at the microsatellite DNA level, COPD patients revealed higher frequency of somatic mutations when compared with asthmatics [45]. In the same study COPD specific microsatellite sites were identified adjacent to genes related to COPD pathogenesis (e.g., Surfactant A, Perforin, CD8, Tumor Necrosis Factor (TNF)). In a recent study Dima et al. [46] found that COPD patients had increased sputum neutrophils, Interleukin-8, and TNF-*α* levels compared to smoking asthmatics [46]. Sputum neutrophilia was inversely correlated with the change in FEV1 in smoking asthmatic patients while no correlation was found between BHR and inflammation in asthmatic patients, regardless of smoking. Reversibility was not correlated with inflammation in COPD patients. However, the response to adenosine 5′monophosphate (AMP) challenge was correlated with sputum neutrophils, concluding that the combination of lung function testing, sputum induction, and BHR reveals information that facilitates the distinction between these diseases [46].

## 5. Immunosenescence and Obstructive Lung Diseases

The ageing process affects the innate and adaptive immune processes, irrespective of the presence of disease, by a phenomenon known as immunosenescence. Neutrophilic inflammation, similar to that noted in COPD and severe noneosinophilic asthma, is present in the airways of older people, as a result of the lifelong antigenic and environmental exposure and age-related changes to innate immunity [47]. 

Studies in older asthmatics have shown age-related changes in innate immunity including lung epithelial cell senescence, increased airway neutrophilia, decreased eosinophil function, decreased levels of cytotoxic natural killer (NK) cells and macrophages [48, 49]. Age-related changes in adaptive immunity include decreased dendritic cell function and a chronic proinflammatory state characterized by increased levels of Inteleukin-1, Interleukin-6, and TNF-*α* [48, 49]. Studies showing a decrease in Interferon-g/Interleukin-4 and Interferon-g/Interleukin-10 after stimulation of NK T cells in older asthmatics support the notion of a transition to a greater T-helper type 2 (Th2) responses with aging [48–50]. 

On the other hand, aging and COPD are characterized by increases in proinflammatory cytokines such as interleukin Interleukin-6 and TNF-*α*, which are implicated in aging-related inflammatory diseases and correlate with degree of obstruction in COPD [51]. There is an age-dependent decline in naive T cells with oligoclonal expansion of CD8(^+^) CD28null T cells from chronic antigenic stimulation, leading to a decline in adaptive immune response. To compensate for this decline there is a paradoxical upregulation of innate immune system resulting in a proinflammatory state [51]. The dysregulated immune system associated with aging results in recruitment and retention of neutrophils, macrophages, and CD4(^+^) and CD8(^+^) T cells in the lungs of smokers with COPD [51, 52]. Such changes may lead to persistent presence of neutrophils in lungs, which in turn may cause emphysematous destruction via exaggerated release of neutrophil elastase. Cigarette smoke may further contribute to epithelial injury by inducing recruitment of neutrophils and macrophages to the lungs, and such cells in the elderly seem to be activated in uncontrolled and pathological ways, releasing more proinflammatory cytokines and amplifying a cascade of ongoing inflammation and parenchymal lung damage [51–53].

As a final point, changes in the lung that occur with aging often mimic those seen in COPD. Furthermore, immunosenescence is strongly associated with persistently activated innate immunity and possibly uncontrolled adaptive immunity thus, becoming even more difficult to differentiate asthma from COPD in the elderly population.

## 6. Allergy and Atopy

Indoor allergen exposure and sensitization are very common in childhood asthma. The prevalence of atopy has been considered to be uncommon in elderly population with asthma [54]. Recent studies suggest that although the prevalence of atopy may be inferior in the elderly, compared with young population, older adults may have a higher incidence of allergen sensitization than previously believed [54].

It is a matter of debate, however, if allergen exposure and sensitization play the same role in the development of asthma in adults as they do in children. Reports from a recent study by Sin et al. [21] observed a higher prevalence of atopy in elderly asthmatic patients when compared with age-matched controls without asthma. Thus, a positive skin test was revealed in 37% of asthmatic versus 8.3% of COPD patients, while asthmatics had also higher serum total IgE levels compared to COPD patients [21]. Furthermore, previous studies reported an increased incidence of allergic history in asthmatics, starting at age 65 or over [24]. Although the mean size of common allergens was greater in asthmatics, Sitkauskiene et al. [55] did not find any difference in positive skin test response between asthmatics and COPD patients, but a significantly raised serum total IgE level was detected in the asthmatic patients [55]. In another study, Huss et al. [54] showed a positive skin test in 74.6% of elderly asthmatics. The results from another study in the elderly population revealed that the presence of cockroach-specific serum IgE was associated with more severe asthma in elderly patients, with increased airway obstruction and hyperinflation [56].

Although available data seem to favor the decline of allergen sensitization with age, the prevalence of allergic sensitizations in the elderly population with respiratory symptoms is substantial enough to warrant evaluation of the atopic condition. This approach will improve diagnosis and could allow better disease management leading to significant reduction in asthma morbidity [24].

## 7. Conclusions

In clinical practice, the differentiation between Asthma and COPD is often difficult to achieve. Especially in elderly patients with long-term asthma, reversibility of airway obstruction is diminished, and a disease pattern similar to COPD may develop. In addition, smoking and ageing increases BHR. Furthermore, the neutrophils are increased, resulting in asthmatics with a COPD phenotype. 

On the other hand, although it is known that COPD is characterized by chronic airflow limitation that is not fully reversible, there is a subgroup of COPD patients with reversibility, which has been associated with increased exhaled nitric oxide and sputum eosinophilia. COPD is often accompanied by BHR, and smoking as well as ageing seems to be risk factors for increasing BHR whereas smoking cessation improves BHR, both in asthma and COPD patients.

The combination of lung function testing, BHR and atopy status, HRCT scans, and the newly developed biological techniques, allowing the assessment of biomarker profiles, could facilitate the distinction between these diseases.

## Figures and Tables

**Table 1:** Main differences between COPD and Asthma.

	COPD	Asthma
Onset	Mid life	Early or Late onset
Risk factors	Smoking	Atopy, irritant exposures, smoking
Symptoms	Slowly progressive	Intermittent
(i) *Breathlessness *	*On moderate/minimal effort*	*On exacerbations*
(ii) *Cough *	*Regularly*	*On exacerbations*
Family history	May be present	May be present
FEV1/FVC ratio	<70%	<70%
FEV1% predicted	<80%	<80%
Bronchodilator response	Absent/present	Present
Inflammatory cells	Neutrophils	Eosinophils
	Macrophages	Neutrophils
	CD8^+^ cells (Tc)	Mast cells
		CD4^+^ cells (Th2)
Structural changes	Airway smooth muscle +	Airway smooth muscle +++
	Peripheral airways	All airways
	Parenchymal destruction	No parenchymal involvement
	Epithelial metaplasia	Epithelial shedding
	Fibrosis++ (peribronchiolar)	Fibrosis + (subepithelial)
	Mucus secretion +++	Mucus secretion +
Steroid response	+/−	+++
